# Influence of Polyplex Formation on the Performance of Star-Shaped Polycationic Transfection Agents for Mammalian Cells

**DOI:** 10.3390/polym8060224

**Published:** 2016-06-06

**Authors:** Alexander Raup, Ullrich Stahlschmidt, Valérie Jérôme, Christopher V. Synatschke, Axel H. E. Müller, Ruth Freitag

**Affiliations:** 1 Process Biotechnology, University of Bayreuth, 95440 Bayreuth, Germany; alexander.raup@uni-bayreuth.de (A.R.); ullrich.stahlschmidt@uni-bayreuth.de (U.S.); valerie.jerome@uni-bayreuth.de (V.J.); 2 Simpson Querrey Institute for BioNanotechnology, Northwestern University, Chicago, 60611 IL, USA; christopher.synatschke@northwestern.edu; 3 Institute of Organic Chemistry, Johannes-Gutenberg-University, 55099 Mainz, Germany; axel.mueller@uni-mainz.de

**Keywords:** gene delivery, mammalian cells, non-viral, PDMAEMA, T lymphocytes, transfection

## Abstract

Genetic modification (“transfection”) of mammalian cells using non-viral, synthetic agents such as polycations, is still a challenge. Polyplex formation between the DNA and the polycation is a decisive step in such experiments. Star-shaped polycations have been proposed as superior transfection agents, yet have never before been compared side-by-side, e.g., in view of structural effects. Herein four star-shaped polycationic structures, all based on (2-dimethylamino) ethyl methacrylate (DMAEMA) building blocks, were investigated for their potential to deliver DNA to adherent (CHO, L929, HEK-293) and non-adherent (Jurkat, primary human T lymphocytes) mammalian cells. The investigated vectors included three structures where the PDMAEMA arms (different arm length and grafting densities) had been grown from a center silsesquioxane or silica-coated γ-Fe_2_O_3_-core and one micellar structure self-assembled from poly(1,2-butadiene)-block PDMAEMA polymers. All nano-stars combined high transfection potential with excellent biocompatibility. The micelles slightly outperformed the covalently linked agents. For method development and optimization, the absolute amount of polycation added to the cells was more important than the N/P-ratio (ratio between polycation nitrogen and DNA phosphate), provided a lower limit was passed and enough polycation was present to overcompensate the negative charge of the plasmid DNA. Finally, the matrix (NaCl *vs.* HEPES-buffered glucose solution), but also the concentrations adjusted during polyplex formation, affected the results.

## 1. Introduction

Various cellular barriers and defense mechanisms challenge the genetic modification of mammalian cells. Viruses have evolved to specifically and efficiently overcome these barriers in order to infect their target cells. Applications that require high efficiency, e.g., human gene therapy, hence tend to use viral delivery strategies, typically based on non-replicating adeno-associated virus (AAV) vectors [1,2]. AAV-based agents can deliver genes to both dividing and non-dividing cells, but the delivered DNA is not integrated into the genome. Gene expression is therefore short lived, even in non-dividing cells. Concomitantly, all viruses elicit an immune response, however small. In certain cases, strong toxic or inflammatory reactions have been observed, as in the infamous Jesse Gelsinger-case [3]. Finally, the challenge of producing large amounts of viruses adds to the costs of such therapeutics.

Non-viral (synthetic) transfection agents are easier to produce at large scale, they can be stored for any length of time, while further modification and customization of their chemical backbone is possible as our understanding of the transfection mechanism evolves [4]. Typically, the first task of the non-viral vector is the compaction and electrostatic neutralization of the negatively charged DNA and the field is therefore dominated by natural and (semi-)synthetic polycations and cationic lipids [5,6,7,8]. However, while certainly safer, [9], non-viral delivery of DNA is currently also much less efficient [10] than viral delivery, in particular where non-dividing, primary, or non-adherent cells are concerned. Consequently, the use of non-viral agents is at present largely restricted to cells from mammalian cells lines intended for the recombinant production of biopharmaceuticals. For such applications non-viral agents perform well, reaching transfection efficiencies of >90% together with stable transgene expression for sometimes well over 50 and more cell generations [11,12]. 

Past research on non-viral transfection agents has given some hints in regard to possible structural improvements. For instance, non-linear polymers appear to be more efficient and less cytotoxic than linear polymers of the same size [6,13,14]. Recently, our group has published first data showing that star-shaped nano-structures with many arms extending from a central core may result in non-viral polycationic transfection agents with the ability to transfect cells, which up to then had been considered unsuitable targets for non-viral gene delivery [15]. Examples include suspension cells (Jurkat cells), growth-arrested and differentiated cells (C2C12 cells) and even primary human T lymphocytes. The nano-stars also performed adequately in the presence of serum, which conventional polycationic agents, including the current ‘gold standard’ PEI (poly(ethyleneimine)) typically will not do [16,17]. 

Whereas previous experiments involving star-shaped structures had focused on their unprecedented ability to deliver DNA to “difficult-to-transfect”-cells [15,18], herein aspects of polyplex formation on performance are discussed, most importantly the transfection matrix and the means of adjusting the N/P-ratio. Moreover, the proposed vectors are for the first time compared in side-by-side experiments using the respective optimized protocols but otherwise identical experimental conditions (cells, plasmid preparations, tools and infrastructure). In this context we evaluated not only the transfection efficiency itself, *i.e.*, the percentage of successfully transfected cells, but also the distribution of the transgene expression level (high, middle, and low producers) over the population as well as the biocompatibility (culture viability) of the various agents and conditions.

## 2. Materials and Methods

### 2.1. Materials

If not otherwise indicated, we used Biochrome AG (Berlin, Germany) and Greiner bio-one (Frickenhausen, Germany) as supplier for cell culture materials, media and solutions and Sigma-Aldrich (Taufkirchen, Germany) for chemicals. Blood, as source for primary human T lymphocytes, was obtained from the Bavarian Red Cross (Nuremberg, Germany, different donors).

### 2.2. Cell Culture Media

The following media and supplements were used. R10 (R15): RPMI-1640 (without l-glutamine, Lonza, Visp, Switzerland) supplemented with 10 vol % (15 vol %) fetal calf serum, 2 mM l-glutamine, and 100 IU/mL penicillin/100 µg/mL streptomycin; MEM10: Minimum Essential Medium Eagle (without l-glutamine and FCS, Biochrome AG, Berlin, Germany) supplemented with 10 vol % FCS, 4 mM l-glutamine, and 100 IU/mL penicillin/100 µg/mL streptomycin; Opti-MEM: reduced serum medium, GlutaMAX™ Supplement (Invitrogen/Life Technology/Thermo Fisher Scientific, Waltham, MA, USA); T lymphocyte medium: Quantum PBL medium (PAA Laboratories, Pasching, Austria). For pre-equilibration, media were incubated for at least 1 h in a standard mammalian cell culture incubator (37 °C, 5% CO_2_, 95% humidity).

### 2.3. Buffers and Solutions

DPBS: Dulbecco’s Phosphate-Buffered Saline without Ca^2+^ and Mg^2+^; NaCl: 150 mM NaCl in Milli Q water (sterilized by filtration, 0.2 µm, cellulose acetate); HBG: 20 mM HEPES, 5 wt % glucose, pH 6.0 (sterilized by filtration).

### 2.4. Plasmid

pEGFP-N1 (4.7 kb, Clontech Laboratories, Inc., Mountain View, CA, USA) encoding for the enhanced green fluorescent protein (EGFP) driven by the cytomegalovirus (CMV) immediate early promoter was used as reporter. pEGFP-N1 was amplified in *E. coli DH5α* using standard laboratory techniques and purified by EndoFree Plasmid Kit (Giga Prep) from QIAGEN (Hilden, Germany). Purified plasmids were solubilized in PCR-water (Sigma-Aldrich). Quality control: > 80% supercoiled topology (agarose gel) and A_260_/A_280_ ≥ 1.8.

### 2.5. Polycationic Transfection Agents

**Figure 1:**
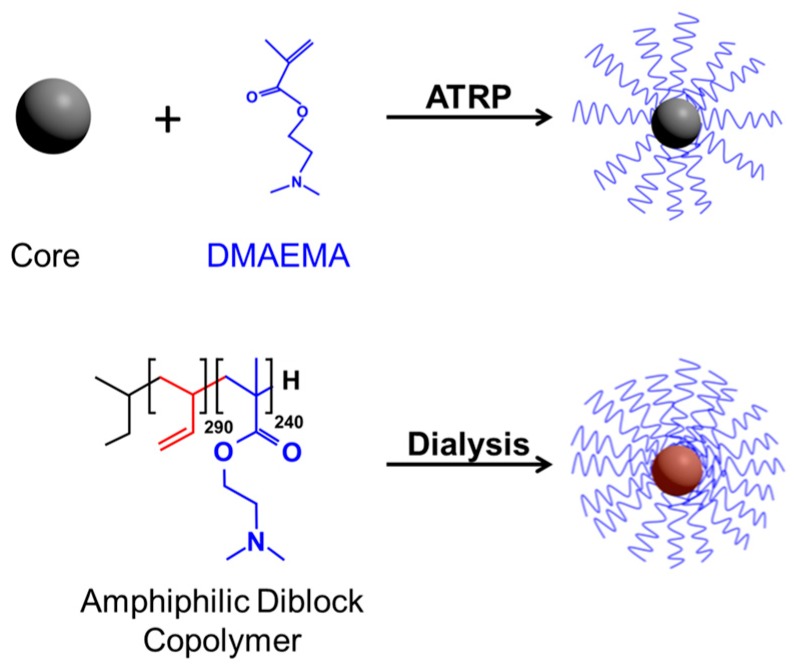
Synthetic procedures for DMAEMA-based Nano-stars. Preparation from multifunctional initiators (Core) via ATRP (**Top**) and self-assembly of amphiphilic diblock copolymer PB290-*b*-PDMAEMA240 to star-shaped micelles (**Bottom**).

All transfection agents considered herein (Table 1) were based on PDMAEMA (poly(2-dimethylamino) ethyl methacrylate) and synthesized in house and characterized as previously published. In particular these were: (PDMAEMA_230_)_20_ [15]: silsesquioxane nanoparticle core; stock solution: 183 mg/mL in sterile Milli Q water (1150 mM N-residues). Poly(1,2-butadiene)-*block*-PDMAEMA (B_290_D_240_) [15,19]: polybutadiene hydrophobic core (290 monomeric units); stock solution: 10.7 mg/mL in sterile PBS (48.3 mM N residues); molecules form star-shaped micelles in aqueous solution. γ-Fe_2_O_3_@silica@(PDMAEMA_540_)_91_ [20]: silica-coated γ-Fe_2_O_3_-nanoparticle core; stock solution: 1.75 mg/mL in sterile Milli Q water (31.1 mM N-residues). In addition, a γ-Fe_2_O_3_@silica@(PDMAEMA_1037_)_46_ (stock solution: 4.5 mg/mL, 19.2 mM N-residues) was prepared analogously to γ-Fe_2_O_3_@silica@(PDMAEMA_540_)_91_. Details on the structures of these agents are schematically shown in Figure 1.

Stock solutions were diluted by sterile Milli Q water as needed. After dilution, an equilibration time of at least 6 h was allowed for prior to use.

### 2.6. N/P-ratio Calculation

N/P-ratios were calculated according to:

$$\text{Number of equivalent}=\frac{\mu L\;\text{polycation stock solution}\times \left[N\right]}{\mu g\;\text{pDNA}\times 3}$$

with [N] = concentration of nitrogen residues in mM.

### 2.7. Cells

Commercially available cell lines CHO-K1 (CCL-61, ATCC), L929 (murine fibroblast, CCL-1, ATCC), HEK-293 cells (CRL-1573, ATCC) and Jurkat (human leukemia T cells, TIB-152, ATCC) were maintained as recommended by the supplier (culture media: R10 for CHO-K1, HEK-293 and Jurkat, MEM10 for L929). Peripheral blood mononuclear cells (PBMCs) were isolated from the blood of healthy donors by Ficoll density gradient centrifugation (lymphocyte separation medium LSM 1077, PAA Laboratories, Pasching, Austria), according to the supplier’s instructions. Interface cells (typically 70%−100% lymphocytes) were washed three times with DPBS (centrifugation: 10 min, 300× *g* followed by gentle resuspension in fresh DPBS) and seeded at 4 × 10^6^ cells/mL in Quantum PBL medium. Note, this medium contains proprietary amounts of phytohemaglutinin (PHA) to selectively stimulate growth of T cells. During cultivation 1/3 of the medium was exchanged daily.

### 2.8. Transfection Protocols

Adherent cells were harvested by trypsinization (standard laboratory protocols including trypsin inactivation by growth medium) and seeded at 2 × 10^5^ cells in 2 mL growth medium in 6-well plates. 1 h prior to transfection, cells were rinsed with DPBS and supplemented with 1 mL of Opti-MEM. Unless indicated otherwise, polyplexes were prepared by mixing up to 3 µg of plasmid DNA in a final volume of 200 µL of the indicated transfection matrix (NaCl or HBG) and the required amount of polycation to reach the N/P-ratio was added in a single drop. After 20 min incubation, the polyplex suspension was diluted with 1 mL Opti-MEM and further incubated for 10 min before being added drop-wise to the cells. After 2 to 4 h incubation, the cells were transferred into fresh growth medium.

Alternatively and in particular in case of the large γ-Fe_2_O_3_-based stars, transfections were carried out in 6-well plates. The DNA (1 µg) was placed in only 50 µL transfection matrix and diluted by 1 mL of Opti-MEM *prior* to the addition of the polycation in a single drop and followed by a 30 min incubation step. The subsequent steps were similar. 

Non-adherent cells were washed once with DPBS (5 min centrifugation, 200× *g*, followed by gently resuspension in DPBS, followed by re-centrifugation) 2 h *prior* to transfection and seeded at 6 × 10^5^ cells per well in 1.0 mL Opti-MEM in 6-well plates or at 3 × 10^5^ cells per well in 0.3 mL Opti-MEM in 24-well plates, as indicated. The polyplex suspension prepared as indicated, was diluted with 0.3 mL (24-well plates) and 1.0 mL (6-well plates) Opti-MEM and added to the cells. The plates were then placed for 4 h in the incubator. Afterwards, half of the medium were removed, taking care not to disturb the cells, and 0.75 mL (24-well plate) or 1.3 mL (6 well plate) of pre-equilibrated fresh growth medium containing 15% FCS were added.

### 2.9. Zeta Potential Measurements

For zeta potential measurements (Zetasizer Nano ZS, Malvern, Herrenberg, Germany) polyplexes of the γ-Fe_2_O_3_-based agents were prepared by mixing 1 µg DNA into 50 µL 150 mM NaCl solution, then adding 1 mL Opti-MEM followed by the respective amount of polycation stock solution (single drop) to reach the N/P-ratio. For (PDMAEMA_230_)_20_ and B_290_D_240_, 15 µg of DNA were placed in 1 mL 150 mM NaCl solution followed by the addition of the required amounts of polycation stock solution in a single drop. Mixtures were vortexed for 10 s and incubated for 30 min at room temperature for polyplex formation and maturation. Zeta-potential measurements were performed in triplicate at room temperature.

### 2.10. Flow Cytometry Analysis

The instrument was a Cytomics FC500 (Beckman Coulter, Krefeld, Germany) equipped with a 488 nm, argon-ion laser. Forward scatter (FSC), side scatter (SSC), green fluorescence (FL1, 525 m) and red fluorescence (FL3, 620 nm) were recorded, the FL1 and FL3 signals on a logarithmic scale. For the measurements, non-adherent cells were recovered by centrifugation (200× *g*, 5 min) and resuspended in DPBS containing 1 µg/mL propidium iodide (PI) to counterstain the dead cells (for the viability measurements, see below). Adherent cells were harvested by trypsinization, pooled with the original culture supernatant to include putative detached (mitotic, dead) cells from the culture, and then treated analogously. Cells were initially evaluated by scatter properties (FSC/SSC) in order to select a region representing single, non-apoptotic cells (elimination of dead cells, debris and cellular aggregates). Data were collected from at least 20,000 events. In addition, the relative EGFP fluorescence of the gated cells was quantified thereby allowing both a statistical quantification of the percentage of transfected cells (“transfection efficiency”) as well as of the expression level distribution according to: low producer: fluorescence intensity between 1 and 10 a.u. (arbitrary units); middle producer: fluorescence intensity between 10 and 100 a.u.; high producer: fluorescence intensity > 100 a.u.) in the non-apoptotic cell population. Concomitantly the total cell population was analyzed for red fluorescence intensity (PI) to determine the overall viability. Negative controls, *i.e.*, non-transfected cells, were used to set the position of the quadrants separating EGFP-positive living cells (upper left), EGFP-positive dead cells (upper right), EGFP-negative living cells (lower left), and EGFP negative dead cells (lower right).

### 2.11. Statistical Analysis

Experiments were performed at least in triplicate and data are presented as mean ± SD. Spearman’s non-parametric rank correlation coefficient (*r*_s_) was used to examine the relationship between N/P ratio or DNA/polycation amount per well and transfection efficiency/viability. Values for the correlation coefficient were scored as a small (*r*_s_ ≤ 0.30), moderate (0.30 ≤ *r*_s_ ≤ 0.7), or strong (*r*_s_ ≥ 0.7). *r*_s_-values for pairs, for which the statistical relevance, *p*, ranked > 0.01 were considered uncorrelated. In this context, for pairs of variables with positive correlation coefficients and *p* < 0.01 both variables increase/decrease together, while for pairs with negative correlation coefficients and *p* < 0.01, one variable increases while the other decreases. All calculations were performed using SigmaPlot (version 11.0) (Systat Software GmbH, Erkrath, Germany).

## 3. Results and Discussion

### 3.1. Zeta Potential Measurements

For transfection, the DNA-containing polyplexes should have a positive net-charge in order to be attracted to the negatively charged cells. A surplus of polycation is therefore required and the parameter most routinely adjusted in non-viral transfection protocols using polycationic agents is the N/P-ratio, *i.e.*, the ratio between the nitrogen residues in the transfection agent and the phosphate units in the DNA. Table 2 summarizes the zeta potentials measured for the different polyplexes as a function of the N/P-ratio as indication of their charge status. 

N/P-ratios in the zeta potential measurements were *inter alia* chosen to include also typical conditions to be expected in the subsequent transfection experiments [21], *i.e.*, in addition to N/P-ratios around the expected “threshold” value for full charge compensation, at least two higher N/P-ratios more typical for actual transfection protocols were considered. In case of (PDMAEMA_230_)_20_ and B_290_D_240_ additional N/P-ratios of 10 and 20 were thus chosen based on previously established protocols [15], while for the less studied γ-Fe_2_O_3_-based constructs the N/P-ratio was increased in smaller steps. 

Depending on the type of nano-star, N/P-ratios between 3 and 7 were required to charge-neutralize the DNA. Hence, the amounts of polycation required for achieving polyplex electroneutrality and by inference the effective charge density of the polycations differed considerably. Moreover, the value for P in these estimations was calculated based on the mass of added DNA, assuming that each base pair contributes 2 charges. While this is most likely correct for linear DNA, it has been shown the effective charge density of plasmid DNA can be considerably lower. Aicart gives values between −0.19 and −0.46 for a plasmid of similar size as the one used by us [22]. A comparison with linear DNA would be required to determine the effective charge density of the pDNA in our case. However, since the same pDNA was used in all experiments, a comparison of the relative effective charges of the different nano-stars becomes possible even in the absence of the absolute values. On the molecular level these differences must be correlated to differences in the ratio of protonated amino groups under conditions for polyplex formation, which in turn must be linked to the polymer structure, since all investigated nano-stars were based on DMAEMA-units. 

The lowest N/P-ratio for complete charge compensation, namely an N/P-ratio of 3, was observed for the micellar agent B_290_D_240_, which presumably also has the most freedom in arranging its structure to interact with the DNA. (PDMAEMA_230_)_20_ and γ-Fe_2_O_3_@silica@(PDMAEMA_540_)_91_ require an N/P-ratio of at least 5, while in case of γ-Fe_2_O_3_@silica@(PDMAEMA_1037_)_46_ the ratio between N and P had to be >5 to overcome the negative DNA charge. The latter observation is of particular interest, since the two γ-Fe_2_O_3_-based nano-stars used in this investigation bore a very similar number of charged units per nano-particle. With 91 arms, the number of arms in γ-Fe_2_O_3_@silica@(PDMAEMA_540_)_91_ was twice as high as that of γ-Fe_2_O_3_@silica@(PDMAEMA_1037_)_46_. To compensate, the arms were only half as long. However, due to the larger center core (Table 1), the arms density of the γ-Fe_2_O_3_@silica@(PDMAEMA_540_)_91_ nano-stars was only 0.035 arms per nm^2^, *i.e.*, roughly half as high as that of γ-Fe_2_O_3_@silica@(PDMAEMA_1037_)_46_ (0.054 arms per nm^2^). For comparison, (PDMAEMA_230_)_20_ has only 20 arms and the highest grafting density (0.71 arms per nm^2^) among the tested polymers and B_290_D_240_ has the highest number of arms and a grafting density of 0.12 arms per nm^2^ (Table 1).

Interpolymer salt bonds of a minimum length are considered necessary for cooperative binding between cationic macromolecules and DNA. Compared to linear polycations, star-shaped polymers have less flexibility to arrange their positively charged groups opposite to the phosphate groups of the rather stiff DNA helix. Moreover, connectivity and spacing between the amine groups influence polycation protonation [23], while electrostatic repulsion between adjacent charged groups may lead to suppression of polycation ionization [24]. Thus, the interrelationship between electrostatic interactions, grafting density and protonation grade of the polycations is complex and may explain the observed differences. An in depth interpretation of these data would require a nano-stars library, in which arm length and arm number are varied systematically. Such an analysis is beyond the scope of this paper.

### 3.2. Adjustment of the N/P-Ratio for Transfection

**Figure 2:**
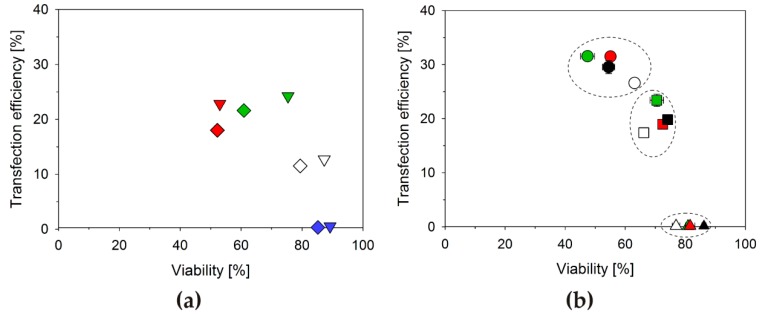
Transfection efficiency and viability as a function of the N/P-ratio and its adjustment. (**a**) Jurkat cells were transfected with B_290_D_240_, using 3 µg DNA/well and increasing the polycation concentration to achieve the N/P-ratio. N/P-ratios: 2.5 (blue, 5.0 µg_(Polymer)_/well), 5 (white, 9.9 µg_(Polymer)_/well), 7.5 (green, 14.9 µg_(Polymer)_/well) and 10 (red, 19.8 µg_(Polymer)_/well). The transfection efficiency was analyzed 20 h (◇) and 40 h (▽) *post* transfection; (**b**) Jurkat cells were transfected with a given amount of B_290_D_240_ and increasing the DNA concentration to achieve the N/P-ratio. N/P-ratios: 5 (white), 7.5 (green), 10 (red) and 20 (black). The amount of polycation per well was 1 µg (triangles), 5 µg (squares), or 10 µg (circles). The transfection efficiency was analyzed 40 h *post* transfection.

The N/P-ratio required for charge compensation represents a lower limit, required for interaction with the cell surface. The N/P-ratio optimal for transfection may be considerably higher and, for a given transfection agent, will most likely also differ from cell line to cell line. The optimization of the N/P-ratio is therefore a routine step in the development of any transfection protocol. Since the goal is the delivery of the DNA to the cells, the standard approach for determining/optimizing the N/P-ratio is to keep the DNA amount constant, while varying (increasing) that of the polycation, typically starting from the concentration sufficient for producing positively charged polyplexes. For our case, the results of such an approach are exemplarily shown in Figure 2a. 

Human leukemia T cells (Jurkat cells) were transfected with B_290_D_240_ as delivery agent at N/P-ratios of 2.5, 5.0, 7.5, and 10.0. The DNA amount was 3 µg per well (24-well plates) in all experiments. Jurkat cells were chosen in these experiments, since they are known to be difficult to transfect and rather sensitive towards polycations. Statistically relevant differences in the results are therefore more easily demonstrated. The results summarized in Figure 2a can then be considered typical: The transfection efficiency (percentage of cells expressing the transgene) increases with increasing N/P-ratio, while the culture viability decreases. The N/P-ratio finally chosen for transfection, thus represent a compromise between transfection efficiencies and cell survival. Based on these results, we would determine an N/P-ratio of 7.5 as being optimal for transfecting the Jurkat cells under such conditions.

Free polycations are known to be considerably more cytotoxic than the corresponding polyplexes [13,25]. Adjusting the N/P-ratio via the polycation concentration, while keeping the DNA amount constant, may therefore not be sufficient to understand the effect of the N/P-ratio in transfection and biocompatibility. In principle the N/P-ratio can also be adjusted via changing the DNA amount, while keeping the total polycation concentration constant, although, as far as we could ascertain this has never been discussed before. Results for such an experiment are shown in Figure 2b. Jurkat cells were again transfected using B_290_D_240_. Incidentally, this set of experiments also allows an interpretation of the effect of changes in the *polyplex* concentration at a given N/P-ratio, as the experiment, e.g., for N/P 5 at 10 µg total polycation amount was obviously done by adding ten times as much of the N/P 5 polyplex preparation to the cells as in the experiment at 1 µg total amount of polycation.

Rather than changing with the N/P-ratio, as in Figure 2a, the data points in Figure 2b are grouped according to polymer concentration. When only 1 µg of polycation was added, high viabilities, but hardly any transfection was observed, *regardless of the N/P-ratio*. The highest transfection efficiencies were consistently found when 10 µg polycation had been added per well to the cells; concomitantly, the lowest culture viabilities were determined in these experiments. However again there is no clear link between the N/P-ratio and the transfection efficiencies (biocompatibilities) in these experiments. Instead, within a group defined by total polycation concentration, the respective highest N/P-ratios *did not* coincide with the lowest viabilities, but rather combined highest transfection efficiency with better than average biocompatibility. This was even maintained when the N/P-ratio in these experiments was driven to the unusually high value of 20.

**Figure 3:**
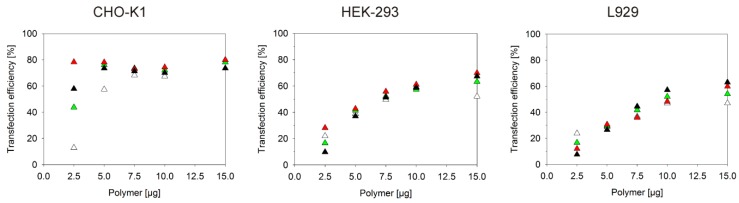
Transfection efficiency as a function of the polymer concentration. CHO-K1, HEK-293 and L929 cells were transfected with B_290_D_240_ at N/P-ratios: 5 (white), 7.5 (green), 10 (red) and 20 (black). The amount of polycation per well was kept constant and the DNA amount adjusted to achieve the N/P-ratio. The transfection efficiency was analyzed 40 h *post* transfection.

Jurkat cells were chosen in these experiments to make the trends more visible. However, similar dependencies were observed for the other cell lines (Figure 3) and transfection agents (Table 3). This is well seen in Figure 3, where transfection efficiencies are plotted against the total polycation concentration added to a particular cell culture sample, rather than the more common plotting against the N/P-ratio. In order to also consider putative effects of the N/P-ratio in this set of experiments, for each polymer amount different N/P-ratios were adjusted via the added DNA. Clearly, transfection efficiencies change as a function of the polycation amount, while the results for the different N/P-ratios per polycation concentration tend to cluster, especially for the higher polycation concentrations. The effect of the polymer concentration on culture viability was more difficult to evaluate in this case, since the reaction of the CHO, HEK, and L929 cells to the added polycations was much less sensitive than that of the Jurkat cells and viabilities consistently remained above 95% throughout these experiments. 

Finally in case of γ-Fe_2_O_3_@silica@(PDMAEMA_1037_)_46_, Spearman’s rank correlation coefficients (*r*_s_) were calculated to correlate transfection efficiencies and viabilities with the N/P ratio, the DNA and the polymer amounts per well, Table 3. γ-Fe_2_O_3_@silica@(PDMAEMA_1037_)_946_ was the nano-star that had not been investigated before, hence the most comprehensive set of data was produced for that molecule, allowing a detailed statistical evaluation. As far as we could ascertain, however, tends were similar for the other investigated polycations.

In all investigated cell lines, the transfection efficiency showed a statistically significant strong correlation with the polymer amount in the well. The correlation with the N/P ratio was also present, but only of moderate magnitude, whereas no significant correlation was found for the DNA concentration per well and the transfection efficiency. For the viabilities, a negative correlation is observed for all three parameters. As to be expected, the polymer amount in the wells has the strongest impact on the viability, followed by the N/P ratio and finally the DNA amount. Moreover, in case of the Jurkat cells only the polymer content correlated with statistical significance to the viability.

Based on these results it can be deduced that the absolute amount of added transfection agent (*i.e.*, polycation) determines the outcome of the experiment, while the amount of DNA or that of the N/P-ratio is of less consequence, provided the latter is high enough to complex and charge neutralize the DNA, *i.e.*, in case of B_290_D_240_ at least N/P = 3. Moreover, this suggests that the established way of setting N/P-ratios in transfection protocols—via changes in the polymer concentration—may have to be reexamined.

### 3.3. Influence of the Transfection Matrix

Size and density of the polyelectrolyte complexes between the DNA and the nano-stars depend on the conditions adjusted during polyplex formation. In turn these parameters may influence polyplex performance during transfection. Van Gaal *et al.* described, e.g., how the matrix provided during polyplex formation has a non-negligible effect on polyplex size distribution and finally on transfection efficiency/biocompatibility [21,26]. In our case the salt concentration, but also the pH was expected to have an influence, since the charge density of PDMAEMA depends on the pH. In the our original standard protocol, however, as well as in most published protocols, an unbuffered 150 mM NaCl-solution was indicated as matrix for polyplex preparation. While this corresponds to physiological osmolarities and suffices to reduce non-specific electrostatic interaction, the pH is not controlled in such mixtures and will vary, in particular in experiments where the N/P-ratio is optimized via increasing the amount of polycation. Working in an unbuffered solution, the N/P-ratio alone is then no longer sufficient to define the system.

**Figure 4:**
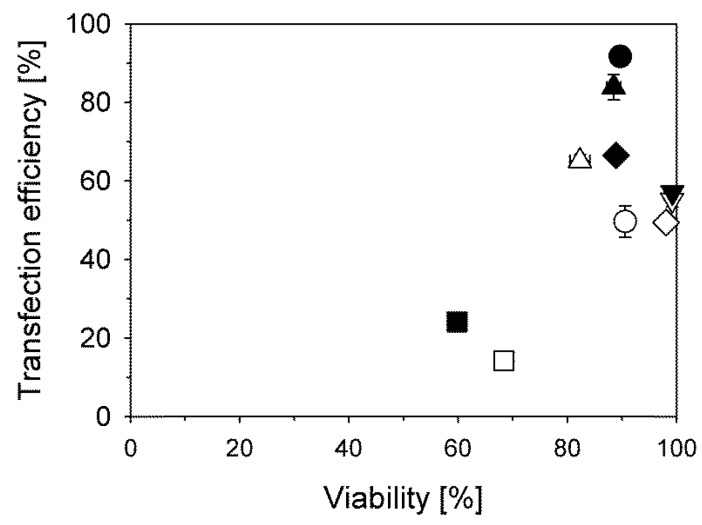
Influence of the polyplex formation matrix (NaCl (empty symbols) *vs.* HBG (filled symbols)) on transfection outcome. Cells (L929 (△), CHO-K1 (◯), Jurkat (☐)) were transfected in 6-well plates (total volume 2.2 mL) with B_290_D_240_ at a N/P-ratio of 7.5 (15 µg polymer per well). CHO cells were in addition transfected with γ-Fe_2_O_3_@silica@(PDMAEMA_540_)_91_ (▽) at a N/P-ratio of 10 (9.6 µg polymer per well) and with (PDMAEMA_230_)_20_ (◇) at a N/P-ratio of 20 (60 µg polymer per well).

When we replaced the non-buffered 150 mM NaCl matrix by the HEPES-buffered glucose solution (HBG) suggested by van Gaal *et al.* [26], transfection efficiencies for the CHO cells increased from 50% to 90% (B_290_D_240_ and (PDMAEMA_230_)_20_) at a similar biocompatibility, while for L929 cells this value increased from 65% to 85% also with a trend towards improved viability, Figure 4. In case of the Jurkat cells, an improvement of the transfection efficiency was also observed, albeit accompanied by a decrease in viability. When the polyplexes were formed directly in the culture medium (Opti-MEM) after dilution of the DNA in the matrix, as for the γ-Fe_2_O_3_@silica@(PDMAEMA_540_)_91_, we could not detect any differences in transfection efficiencies (CHO cells) showing that for the star-shaped polymers investigated here, the ionic strength most likely plays a crucial role for the transfection outcome. This has been discussed before for linear PDMAEMA [21].

**Figure 5:**
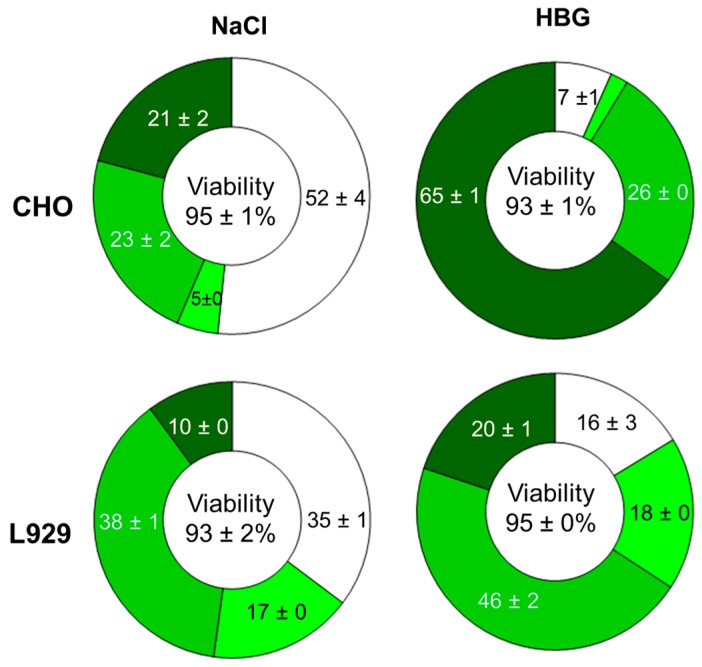
Effect of the polyplex formation matrix (NaCl *vs.* HBG) on the distribution of the transgene expression level within the populations. Cells were divided into: non-producers (white, fluorescence signal < 1 a.u.), low producers (light green, fluorescence signal between 1 and 10 a.u.), middle producers (green, fluorescence signal between 10 and 100 a.u.), and high producers (dark green, fluorescence signal > 100 a.u.).

In addition to overall the transfection efficiencies and biocompatibilities, the transgene expression level (distribution over high, middle, and low producers) was positively influenced by the change in the transfection matrix. As shown in Figure 5, significantly more ‘high producers’ were created when polyplexes prepared in HBG were used to transfect the cells. Beside the above mentioned non-negligible effect of the transfection medium on polyplex size distribution and thus on transfection efficiency/biocompatibility [21], our data suggest that the polyplex size and/or particle stability might also affect the quantity of active DNA which reaches the nuclei. Several previous reports comment on a possible influence of the compactness of the polyplex structures on the performance of polyplexes as transfection agents. In particular, the particle size has been shown to affect the internalization pathway whereas compact structures positively influence DNA protection and cytoplasmic transportation [23,27,28].

### 3.4. DNA Preparation for Polyplex Formation

**Figure 6:**
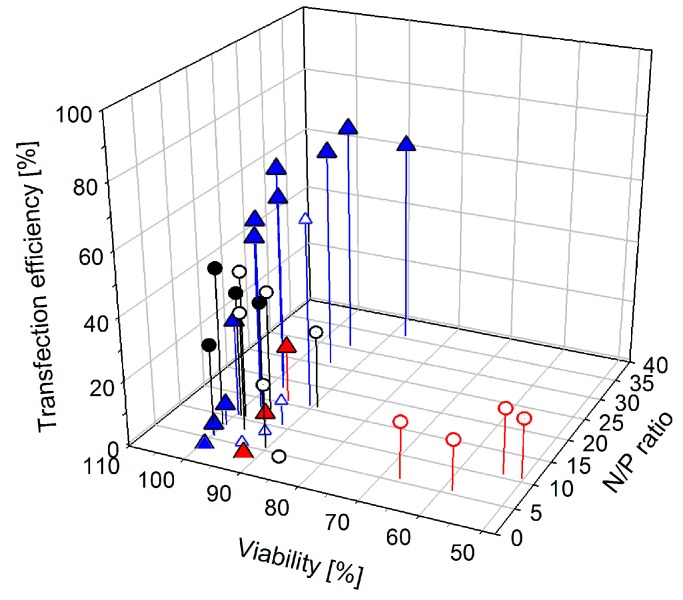
Transfection efficiencies and viabilities as a function of the polyplex preparation protocol for various N/P-ratios. L929 (△) and CHO-K1 cells (◯) were transfected with γ-Fe_2_O_3_@silica@(PDMAEMA_540_)_91_ (empty symbols) and γ-Fe_2_O_3_@silica@(PDMAEMA_1037_)_46_ (full symbols); black and blue symbols: polyplexes formed after initial dilution of the DNA in 1 mL Opti-MEM prior to the addition of the polycation (1 µg DNA; total volume: 2.2 mL), red circles: polyplexes formed according to the standard protocol (3 µg DNA in 200 µL 150 mM NaCl; total volume: 2.2 mL), red triangles: polyplexes formed by incubating DNA with the polycation prior to dilution with 1 mL Opti-MEM (1 µg DNA in 50 µL 150 mM NaCl; total volume: 2.2 mL). All transfections were performed in 6-well plates.

According to most standard protocols including our own, the polyplexes should be prepared in the smallest possible volume, *i.e.*, at the highest possible concentration. As a result the final volume added to the cells is small, presumably causing a minimum of stress. However, when we compared results obtained for the standard protocol (3 µg DNA in 200 µL 150 mM NaCl-solution, addition of the required amount of polycation in a single drop) with those from a protocol where instead the polyplexes were directly prepared in 1 mL Opti-MEM culture medium, we found that for most cell types, biocompatibilities but also transfection efficiencies improved significantly. In this case, the DNA was directly dissolved in 1 mL serum-free Opti-MEM culture medium, followed by the addition of the polycation in a single drop to initiate polyplex formation. The effect was most pronounced in case of the large γ-Fe_2_O_3_-based nano-stars. These agents achieved only low transfection efficiencies (<10%) when the polyplexes were prepared in a small volume and diluted afterwards, while they performed well when the polyplexes were prepared directly in culture medium, Figure 6. Changes in the DNA condensation kinetics and the subsequent particle formation are most likely responsible for the observed improvements.

### 3.5. Direct Comparison for the Transfection of Adherent and Non-Adherent Mammalian Cells

Previous publications on the different star-shaped transfection agents had focused on one particular structure at a time, which makes a comparative evaluation of their performance difficult. Here the three previously published nano-stars, namely (PDMAEMA_230_)_20_, B_290_D_240_, and γ-Fe_2_O_3_@silica@(PDMAEMA_540_)_91_, are for the first time compared side-by-side, using optimized protocols as indicated, but under otherwise identical experimental conditions (cells from one batch, one plasmid preparation, *etc.*). Table 4 summarizes the results obtained for the two adherent (CHO, L929) and the two non-adherent (Jurkat, T lymphocytes) cell types employed in our investigation. Results for the second γ-Fe_2_O_3_-based nano-star specifically prepared for this investigation, namelyγ-Fe_2_O_3_@silica@(PDMAEMA_1037_)_46_, are also given. 

Transfection efficiencies for (PDMAEMA_230_)_20_ were in the same order or even slightly improved compared to the previously published results for CHO, Jurkat, and T cells [15], while the transfection of the L929 cells, not investigated before, posed no difficulty, corroborating our hypothesis that star-shaped cationic nanoparticles in general constitute superior transfection agents for mammalian cells. The same is true for B_290_D_240_, which had previously been used to transfect Jurkat [15], as well as CHO-K1 and L929 cells [18], and which was used here for the first time with a minimum of optimization effort to transfect primary human T lymphocytes. Previous experiments with B_290_D_240_ had already hinted at a superior performance, the direct comparison shows that indeed this non-covalently assembled transfection agent consistently combines the highest transfection efficiencies with excellent biocompatibility. Whether this superior performance is directly linked to the non-covalent nature of the agent, which may, e.g., facilitate transmembrane or intracellular transport or even DNA release, requires further investigation. 

Compared to both (PDMAEMA_230_)_20_ and B_290_D_240_ the performance of the two γ-Fe_2_O_3_-based transfection agents was inferior. It is possible that this was linked to the much bigger size (*i.e.*, molecular weight, for values see Table 1) of these agents, although the biocompatibility, which is normally most strongly affected by size, no longer presented a problem once the protocol for polyplex formation had been modified to using a DNA already diluted in culture medium for polyplex formation. Whereas γ-Fe_2_O_3_@silica@(PDMAEMA_540_)_91_ had been used before to transfect both CHO and L929 cells [20], γ-Fe_2_O_3_@silica@(PDMAEMA_1037_)_46_, *i.e.*, a nano-star bearing a similar number of monomeric units in its arms, albeit at only half the density and twice the arm length, was newly introduced here for comparison. For both CHO and L929 cells, higher transfection efficiencies were obtained for this γ-Fe_2_O_3_@silica@(PDMAEMA_1037_)_46_ than for γ-Fe_2_O_3_@silica@(PDMAEMA_540_)_91_. Elucidating the reason for this difference would require additional experiments, further modifying the basic structure. Our current hypothesis is that the superior performance of γ-Fe_2_O_3_@silica@(PDMAEMA_1037_)_46_ is related to the higher arm density, while the greater length of the arms concomitantly increases flexibility. The improvement in performance could then be due to a similar effect as for the micellar structure B_290_D_240_.

## 4. Conclusions

Star-shaped polycationic nanostructures and in particular micellar ones, are powerful transfection agents for a variety of mammalian cells including difficult-to-transfect suspension and primary cells. Contrarily to common practice, the N/P-ratio in the transfection protocols should be adjusted via the DNA content of the polyplexes rather than the polycation content to avoid bias of the results by the polycation cytotoxicity. Rather than using the smallest possible amount of 150 mM unbuffered saline solution for the preparation of the polyplexes, better results in terms of biocompatibility, may be achieved by preparing the polyplexes in buffered solution such as HEPES-buffered glucose or even directly in the culture medium. Even in the case of cells that already transfect well (>90%) with the standard protocol, such as CHO cells, further improvement is possible, as the range of transgene expression can be shifted towards more high producers, when buffered matrices or culture media are used to prepare the polyplexes.

## Figures and Tables

**Table 1:** Properties of the polymeric transfection agents.

Polymer	*M*_n_ (kDa)	Monomeric units	Arms	Core diameter (nm)	Core surface (nm^2^)	Grafting density (arm/nm^2^)	PDI
(PDMAEMA_230_)_20_	730	230	20	3	28.3	0.71	1.42
B_290_D_240_	54	240	120	18.2	1,041	0.12	1.07
γ-Fe_2_O_3_@silica@-(PDMAEMA_540_)_91_	7,735	540	91	28.8	2,600	0.035	1.38
γ-Fe_2_O_3_@silica@-(PDMAEMA_1037_)_46_^1^	7,498	1,037	46	15.4	850	0.054	1.6

^1^ After synthesis, the nanoparticles were characterized, as previously described [20], by thermogravimetric analysis (TGA) to calculate number and length of the PDMAEMA arms. The core diameter was determined via transmission electron microscopy (TEM) and size exclusion chromatography (SEC). PDI: polydispersity index.

**Table 2:** Zeta potentials of polyplexes prepared at the indicated N/P-ratios.

N/P ratio	(PDMAEMA_230_)_20_	B_290_D_240_	γ-Fe_2_O_3_@silica@-(PDMAEMA_540_)_91_	γ-Fe_2_O_3_@silica@-(PDMAEMA_1037_)_46_
3	−30.7 ± 2.1 mV	0.0 ± 0.1 mV	−9.78 ± 2.41 mV	−8.84 ± 1.29 mV
5	−0.4 ± 4.0 mV	7.3 ± 1.0 mV	−0.24 ± 0.09 mV	−1.99 ± 0.51 mV
7.5	n.d.	n.d.	4.28 ± 0.18 mV	2.95 ± 0.00 mV
10	7.4 ± 1.8 mV	8.8 ± 1.3 mV	5.52 ± 1.01 mV	5.13 ± 0.73 mV
12	n.d.	n.d.	6.73 ± 0.64 mV	6.74 ± 0.32 mV
20	10.1 ± 1.2 mV	10.5 ± 0.6 mV	n.d.	n.d.

(PDMAEMA_230_)_20_- and B_290_D_240_-based polyplexes were prepared using 15 µg DNA in 1 mL of 150 mM NaCl-solution. γ-Fe_2_O_3_@silica@(PDMAEMA_540_)_91_- and γ-Fe_2_O_3_@silica@(PDMAEMA_1037_)_46_-based polyplexes were prepared using 1 µg DNA in 50 µL of 150 mM NaCl-solution followed by dilution in 1 mL Opti-MEM. The zeta potential of the non-complexed DNA is: −26.9 ± 3.2 mV (for 15 µg DNA) and −12.2 ± 1.2 mV (for 1 µg DNA). n.d.: not determined.

**Table 3:** Relationship between transfection efficiency/viability and N/P ratio, DNA and polymer quantities per well estimated by Spearman’s rank correlation coefficient.

Cell line		Transfection efficiency	Viability
CHO-K1	DNA	0.450 (*p* = 0.0114)	−0.527 (*p* = 0.0025)
	Polymer	0.834 (*p* < 0.0001)	−0.923 (*p* < 0.0001)
	N/P ratio	0.662 (*p* < 0.0001)	−0.697 (*p* < 0.0001)
HEK-293	DNA	0.350 (*p* = 0.0365)	−0.466 (*p* = 0.0044)
	Polymer	0.778 (*p* < 0.0001)	−0.656 (*p* < 0.0001)
	N/P ratio	0.601 (*p* < 0.0001)	−0.436 (*p* = 0.0082)
Jurkat	DNA	0.113 (*p* = 0.604)	−0.221 (*p* = 0.308)
	Polymer	0.809 (*p* < 0.0001)	−0.763 (*p* < 0.0001)
	N/P ratio	0.630 (*p* = 0.00127)	−0.471 (*p* = 0.023)

The polymer was γ-Fe_2_O_3_@silica@(PDMAEMA_1037_)_46_.

**Table 4:** Transfection efficiencies and viabilities obtained as a function of the cell type and the transfection agent.

	(PDMAEMA_230_)_20_^c^	B_290_D_240_^c^	γ-Fe_2_O_3_@silica@-(PDMAEMA_540_)_91_^d^	γ-Fe_2_O_3_@silica@-(PDMAEMA_1037_)_46_^d^
**CHO** ^a^				
TE [%]	74.1	91.7 ± 0.6	56.9 ± 0.8	71.4 ± 4.1
Viability [%]	84.4	89.7 ± 1.1	98.5 ± 0.3	95.2 ± 1.8
N/P-ratio [-/-]	20	7.5	10	20
µg_(Polymer)_/well	60	15	9.6	14.0
µg_(DNA)_/well	6.3	3	1	1
**L929** ^a^				
TE [%]	81.6 ± 1.3	87.8 ± 0.5	61.1 ± 2.5	71.0 ± 2.3
Viability [%]	80.2 ± 0.3	91.8 ± 2.2	89.9 ± 1.9	96.8 ± 0.1
N/P-ratio [-/-]	20	10	15	17.5
µg_(Polymer)_/well	80	20	14.5	12.3
µg_(DNA)_/well	8.4	2.9	1	1
**Jurkat** ^b^				
TE [%]	51.6 ± 0.6	44.7 ± 1.3		24.0 ± 0.4
Viability [%]	60.2 ± 4.7	71.3 ± 2.7		56.0 ± 13.0
N/P-ratio [-/-]	20	7.5		25
µg_(Polymer)_/well	19.1	10		8.8
µg_(DNA)_/well	2	2		0.5
**T cells** ^b^				
TE [%]	12.7 ± 0.6	14.6 ± 0.1		
Viability [%]	81.3 ± 0.9	80.1 ± 0.7		
N/P-ratio [-/-]	20	7.5		
µg_(Polymer)_/well	9.5	4.8		
µg_(DNA)_/well	1	1		

^a^: Protocol for adherent cells. ^b^: Protocol for non-adherent cells ^c^: Polyplexes prepared in HBG. ^d^: Polyplexes prepared in 150 mM NaCl (γ-Fe_2_O_3_-based stars). *n* ≥ 2. TE: Transfection efficiency.
